# A review of the differences and similarities between generic drugs and their originator counterparts, including economic benefits associated with usage of generic medicines, using Ireland as a case study

**DOI:** 10.1186/2050-6511-14-1

**Published:** 2013-01-05

**Authors:** Suzanne Dunne, Bill Shannon, Colum Dunne, Walter Cullen

**Affiliations:** 1 Graduate Entry Medical School, University of Limerick, Limerick, Ireland; 2 Centre for Interventions in Infection, Inflammation & Immunity (4i), Graduate Entry Medical School, University of Limerick, Limerick, Ireland

**Keywords:** Generic, Medicine, Drug, Pharmaceutical, Biosimilar, Prescribing, Healthcare, Economics, Ireland

## Abstract

Generic medicines are those where patent protection has expired, and which may be produced by manufacturers other than the innovator company. Use of generic medicines has been increasing in recent years, primarily as a cost saving measure in healthcare provision. Generic medicines are typically 20 to 90% cheaper than originator equivalents. Our objective is to provide a high-level description of what generic medicines are and how they differ, at a regulatory and legislative level, from originator medicines. We describe the current and historical regulation of medicines in the world’s two main pharmaceutical markets, in addition to the similarities, as well as the differences, between generics and their originator equivalents including the reasons for the cost differences seen between originator and generic medicines. Ireland is currently poised to introduce generic substitution and reference pricing. This article refers to this situation as an exemplar of a national system on the cusp of significant health policy change, and specifically details Ireland’s history with usage of generic medicines and how the proposed changes could affect healthcare provision.

## Review

### Summary Introduction: What are Generic Medicines?

Generic medicines are those where the original patent has expired and which may now be produced by manufacturers other than the original innovator (patent-holding) company. The term “generic drug”^a^ or “generic medicine” can have varying definitions in different markets, however the term is commonly understood, as defined by the World Health Organisation (WHO), to mean a pharmaceutical product which:

– is usually intended to be interchangeable with an innovator product,

– is manufactured without a licence from the innovator company, and

– is marketed after the expiry date of the patent or other exclusive rights [1].

There are differing legal requirements in different jurisdictions that define the specifics of what a generic medicine is. However, one of the main principles underpinning the safe and effective use of generic medicines is the concept of bioequivalence.

Bioequivalence has been defined as follows: *two pharmaceutical products are bioequivalent if they are pharmaceutically equivalent and their bioavailabilities (rate and extent of availability) after administration in the same molar dose are similar to such a degree that their effects, with respect to both efficacy and safety, can be expected to be essentially the same. Pharmaceutical equivalence implies the same amount of the same active substance(s), in the same dosage form, for the same route of administration and meeting the same or comparable standards*[2].

The purpose of establishing bioequivalence is to demonstrate equivalence between the generic medicine and the originator medicine in order to allow bridging of the pre-clinical and clinical testing performed on the originator drug.

The objective of this article is to provide an accessible resource describing the foundation of generic medicines, from their legal advent in the mid 1980’s to how current legislation and regulation of generics affects, *inter alia*, their composition, regulatory approval, pricing, and ultimately acceptance by healthcare professionals and patients. In this paper, we also focus on Ireland’s emerging policy on generic medicine use. Ireland is one of the EU ‘bail-out’ countries, and is attempting to conserve resources given the prevailing economic climate. Ireland is, therefore, currently poised to make the legislative changes necessary to introduce generic substitution and reference pricing in order to achieve reductions in the medicines bill for the state. This article refers this situation as an exemplar of a national system on the cusp of significant medical policy change, and specifically details Ireland’s history with usage of generic medicines and how the proposed changes could affect healthcare provision.

## Brief Comparison of Generic Medicines in the United States and Europe

### Generic Medicines in the United States of America

The US Food and Drug Administration [FDA], which regulates the pharmaceutical market in the United States [3] defines generic medicines as:

–*a drug product that is comparable to brand/reference listed drug product in dosage form, strength, route of administration, quality and performance characteristics, and intended use*[4]

–*copies of brand-name drugs and are the same as those brand name drugs in dosage form, safety, strength, route of administration, quality, performance characteristics and intended use*[5].

**Figure 1:**
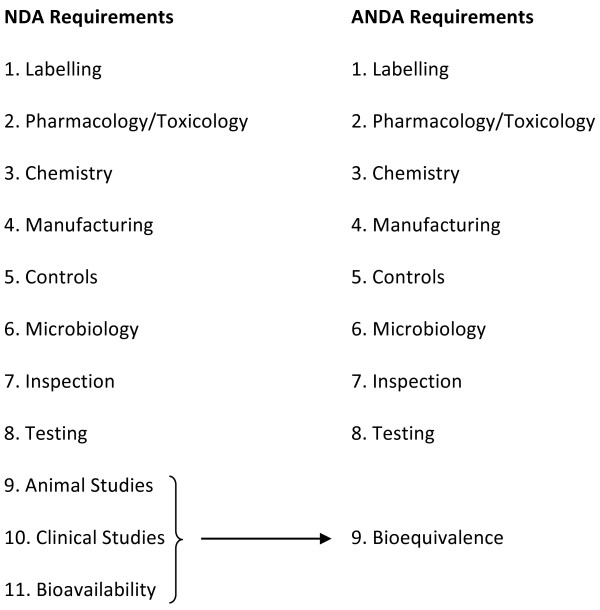
Originator (NDA) versus Generic (ANDA) Review Process Requirements.

The 1984 *Drug Price Competition and Patent Term Restoration Act* (more commonly known as the *Hatch-Waxman Act*) in the US allowed for an abbreviated system for approval of generic copies of all drugs approved after 1962 [4], meaning that pre-clinical and clinical testing did not have to be repeated for generics [6]. The intended result of this legislation was to ensure that generic medicines would be less expensive than the equivalent originator medicine because it was not necessary for generic medicine manufacturers to repeat discovery, pre-clinical and clinical studies [7,8]. (It should be noted, as will be discussed later, that the cost of generics may vary considerably across countries depending on the specific active molecule involved, such that savings may not necessarily always accrue [9]). See Figure 1: **Originator (New Drug Application - NDA) versus Generic (Abbreviated New Drug Application - ANDA) Review Process Requirements**[8] for a comparison of the originator versus generic medicine review process.

To gain FDA approval, a generic medicine must:

- Contain the same active ingredient as the originator medicine (inactive ingredients may vary)

- be identical in strength, dosage form, and route of administration

- have the same use indications

- be bioequivalent

- meet the same batch requirements for identity, strength, purity, and quality

- be manufactured under the same strict standards of FDA's good manufacturing practice regulations required for originator products [10].

Bioequivalence is demonstrated when the rate and extent of absorption do not show a significant difference from the originator drug, or where the extent of absorption does not show a significant difference and any difference in rate is intentional or not medically significant [4]. The FDA’s formal definition of bioequivalence is: *the absence of a significant difference in the rate and extent to which the active ingredient or active moiety in pharmaceutical equivalents or pharmaceutical alternatives becomes available at the site of drug action when administered at the same molar dose under similar conditions in an appropriately designed study*[11]. Therefore, bioequivalent drugs are pharmaceutical equivalents whose rate and extent of absorption are not statistically different when administrated to patients or subjects at the same molar dose under similar experimental conditions [12].

Currently, bioequivalence limits in use by the FDA when assessing a new generic medicine are that the generic medicine demonstrates 80-125% of the bioavailability of the originator drug [12]. In the US, the limit of 80-125% is unchanged for Narrow Therapeutic Range [NTR] drugs [12]. In other countries this is not the always case. In Australia, for example, there are no generic versions of digoxin or phenytoin [both having narrow therapeutic index and bioavailability problems] and, additionally, there are two brands of warfarin on the Australian market which are not considered interchangeable with each other – as no formal bioequivalence comparison of them has been made [2]. However, where branded warfarin was compared to bioequivalent generic formulations, similar outcomes for patients were observed, indicating that brand name warfarin was not superior to a generic alternative in a clinical setting [13].

The bioequivalence limits may suggest that a variance of 25% between an originator brand and a generic product is possible. However, this may not actually be the case. A study was performed which investigated 12 years of bioequivalence data submitted to the FDA, comparing the generic and originator measures from 2070 single-dose clinical bioequivalence studies of orally administered generic medicine products approved by the Food and Drug Administration (FDA) from 1996 to 2007. This study showed that the average difference in absorption into the body between the generic and the originator was 3.5% and is comparable to differences between two different batches of an originator drug [14]. However, it should be noted that variations between batches of originator drugs may themselves threaten patient safety. In 2012, Patel *et al.* reported that (in 2010) patients prescribed Lamotrigine (LTG, an anti-epileptic medication) experienced unexplained toxicity [15]. When investigated, the manufacturer (GlaxoSmithKline) accepted responsibility for an altered formulation due to changes made to the manufacturing process.

### Generic Medicines in the European Union

The legal situation regarding authorisation of pharmaceutical products in the EU is more complex than in the US, with each member state having a competent authority in addition to the European Medicines Agency [EMA], which oversees EU-wide authorisation of medicines.

The EMA defines a generic medicine as: *a medicine that is developed to be the same as a medicine that has already been authorised (the ‘reference medicine’). A generic medicine contains the same active substance(s) as the reference medicine, and it is used at the same dose(s) to treat the same disease(s) as the reference medicine. However, the name of the medicine, its appearance (such as colour or shape) and its packaging can be different from those of the reference medicine*[16]*.*

Authorisation of a medicine in the EU can be done via three different routes: the Centralised Procedure [CP], the Decentralised Procedure [DCP] or the Mutual Recognition Procedure [MRP] [17]. Additionally, National Procedures [NP] are in place in individual member states, which allow a medicine to be authorised by the competent authority in that specific member state.

The **CP**, which came into operation in 1995, allows applicants to obtain a marketing authorisation that is valid throughout the EU. It is compulsory for medicinal products manufactured using biotechnological processes, for orphan medicinal products and for human medicine products containing a new active substance which was not authorised in the Community before 20 May 2004 (date of entry into force of Regulation (EC) No 726/2004) and which are intended for the treatment of AIDS, cancer, neurodegenerative disorder or diabetes. The centralised procedure is also mandatory for veterinary medicinal products intended primarily for use as performance enhancers in order to promote growth of treated animals or to increase yields from treated animals [18]. CP applications are made to, and approved by, the EMA.

To be eligible for the **MRP**, a medicinal product must have already received a marketing authorisation in one Member State. Since 1 January 1998, the MRP is compulsory for all medicinal products to be marketed in a Member State other than that in which they were first authorised. Any national marketing authorisation granted by an EU Member State's national authority can be used to support an application for its mutual recognition by other Member States [19]. The MRP is based on the principle of mutual recognition, by EU Member States, of their respective national marketing authorizations. An application for mutual recognition may be addressed to one or more Member States. The applications submitted must be identical and all Member States must be notified of them. As soon as one Member State decides to evaluate the medicinal product (at which point it becomes the "Reference Member State"), it notifies this decision to other Member States (which then become the "Concerned Member States") to whom applications have also been submitted. Concerned Member States will then suspend their own evaluations, and await the Reference Member State's decision on the product. This evaluation procedure - undertaken by the Reference Member State - may take up to 210 days and, if successful, results in the granting of a marketing authorisation in that Member State. When the assessment is completed, copies of the report are sent to all Member States. The Concerned Member States then have 90 days to recognise the decision of the Reference Member State. National marketing authorizations are granted within 30 days after acknowledgement of the agreement [19].

The **DCP** is similar to the MRP but the difference lies in that it applies to medicinal products that have not received a marketing authorisation at the time of application. With the DCP, an identical application for marketing authorisation is submitted simultaneously to the competent authorities of the Reference Member State and of the Concerned Member States. At the end of the procedure, the product dossier, as proposed by the Reference Member State, is approved. The subsequent steps are identical to the mutual recognition procedure [20].

As in the US, applicants for a marketing authorisation [MA] for a generic medicine in the EU may submit an abbreviated application. According to Article 10(1) of Directive 2001/83/EC [21], an applicant for an authorisation to market a generic medicine is not required to provide the results of pre-clinical and clinical trials if it can be demonstrated that the medicinal product is:

A generic medicinal product or a similar biological medicinal product of a reference medicinal product, which has been authorised under Article 6 of Directive 2001/83/EC for not less than 8 years. This type of application refers to information that is contained in the dossier of the authorisation of the reference product. This information is generally not completely available in the public domain. Authorisations for generic or similar biological medicinal products are therefore linked to the ‘original’ authorisation. This does not however mean that withdrawal of the authorisation for the reference product leads to the withdrawal of the authorisation for the generic product (case C-223/01, AstraZeneca, judgment of the European Court of Justice of 16 October 2003).

*The generic or similar biological medicinal product, once authorised, can however only be placed on the market 10 or 11 years after the authorisation of the reference medicinal product, depending on the exclusivity period applicable for the reference medicinal product*[22].

Generic medicine applications typically include chemical-pharmaceutical data and the results of bioequivalence studies, which demonstrate the similarity of the generic product relative to the reference medicine. As stated previously, the tolerance levels involved have been favourably compared to those acceptable for inter-batch variation during production of the originator medicine [14]. The authorising regulatory agency(ies) is referred to the data that were established in the originator product’s application for authorisation for information concerning the safety and efficacy of the active molecule. This is only possible once the data exclusivity period has expired on the originator product’s dossier [21,23]. The majority of authorizations for generic medicines are granted through the MRP and the DCP. Since the introduction of the DCP, the MRP has mainly been used for extending the existing marketing authorisation to other countries in what is known as the “repeat use” procedure [23].

EU bioequivalence parameters are similar to those mandated in the US, requiring that the test and reference products be contained within an acceptance interval of 80.00 – 125.00% of the AUC [area under the concentration time curve], which reflects the extent of exposure, or C_max_, at a 90% confidence interval. European guidelines, however, also provide a tightened acceptance interval of 90.00-111.11% for narrow therapeutic index drugs [NTIDs] as well as different assessment requirements for highly variable drug products [HVDPs] [24].

Overall, both EU and US legislation for the authorisation of generic medicines allow for abbreviated applications to be made in the case of generic medicines. In both jurisdictions, pre-clinical and clinical studies do not have to be performed by the generic medicine applicant, but bioequivalence to the originator or “reference” medicine must be demonstrated. This abbreviated application process is often quoted as one of the main reasons for the price difference between generic and originator drugs. However, there is variation in generic medicine prices (e.g., within the single market European Union) unrelated to Research and Development expenditure and greatly influenced by local regulations and reimbursement arrangements that may, in some cases, be disassociated from the costs of manufacture and distribution [9]. Other important influencers include demand-side pressures (i.e., education, engineering, economics, and enforcement), International Nonproprietary Name (INN) prescribing, and, specifically, reference pricing which have been widely adopted by European governments [25-29]. It is also worth noting that generic medicine pricing is being driven further downwards as a result of keen competition in this sector. There is evidence of European generic medicine manufacturers facing competition from Indian producers, and a now-established practice of discounting prices to Governments [9,30]. Indeed, experts are now recommending that “European countries must continue learning from each other to fund increased volumes” and so exploit such discounts for bulk purchases [29]. As a result of these and other factors, generic medicines are generally between 20 to 90% cheaper than their originator equivalents [31] which has obvious implications for healthcare costs. For example, in October 2010 in the UK, generic simvastatin (a cholesterol-lowering medicine) cost £1.12 for a pack of 28 (20mg) compared with approximately £30 for a pack of 28 (20mg) of the originator product [32].

## A Brief History of Pharmaceutical Regulations

### Major Pharmaceutical Legislation in the United States of America

Notable regulations published relating to pharmaceutical regulation in the 20^th^ century began in 1906 with the Pure Drug and Cosmetic Act [PDCA] in the United States. In 1905, a book called *The Jungle* was published, in which Upton Sinclair wrote about the Chicago meat packing industry. The book described the unsanitary conditions in which animals were slaughtered and processed, including the practice of selling rotten or diseased meat to the public [33]. This book had a major impact on the American people and led the US Congress to pass the PDCA. With this new law, it became illegal to sell contaminated [adulterated] food or meat, and for the first time labelling of food and drugs had to be truthful – meaning that false or exaggerated claims could no longer be made on labels. The Act also required selected dangerous ingredients to be labelled on all drugs and inaccurate or false labelling was called “misbranding” and also became illegal.

The US Congress passed the Federal Food, Drug and Cosmetic Act [FDCA] in 1938 to complement the PDCA. This was largely in response to a public health disaster with a medicine called Elixir Sulfanilamide in 1937. Elixir Sulfanilamide was a sulfa drug sold as an anti-infective. Over 100 people died, most of them children, following ingestion of this medicine due to the fact that it contained diethylene glycol [DEG] as a solvent. DEG is a chemical analogue of antifreeze and is toxic to humans. The company that manufactured the medicine did not perform any toxicity testing prior to marketing the drug as, at the time, there were no regulations requiring the pre-marketing safety testing of new medicines. The FDCA required, *inter alia*, that new drugs be demonstrated as safe to humans before marketing [34].

The Public Health Service [PHS] Act, which was passed in 1944, was the legal basis for the licensing and gaining of marketing approval of biologic products [35]. Biological products are medicinal products that include vaccines, blood and blood components, allergenics, somatic cells, gene therapy, tissues, and recombinant therapeutic proteins. Biologics can be composed of sugars, proteins, or nucleic acids or complex combinations of these substances, or may be living entities such as cells and tissues. Biologics are isolated from a variety of natural sources - human, animal, or microorganism - and may be produced by biotechnology methods and other cutting-edge technologies. Gene-based and cellular biologics, for example, are often at the forefront of biomedical research, and may be used to treat a variety of medical conditions for which no other treatments are available [36].

The 1962 Kefauver-Harris Drug Amendments [KHDA] added further protection to public health. The KHDA added the requirement that drugs be proven effective for their intended use. With both the 1938 and 1962 laws in place, US regulators were now ensuring that drugs made available to the American public were relatively safe to consume, in addition to being proven effective in treating the disease or condition that they were being marketed in relation to.

In response to the emerging AIDS crisis in the 1980’s, the Orphan Drug Act [ODA] was enacted in 1983 to encourage the development of medicines for conditions that affected small populations by providing monetary and marketing incentives to drug manufacturers. The following year, in 1984, the US Congress also enacted the Hatch-Waxman Act [HWA], which provided for the marketing of generic medicines, the aim of which was to save Americans money on their medicine bills.

The Biologics Price Competition and Innovation Act of 2009 (BPCI Act) was signed into law, by President Barack Obama, on March 23, 2010. The BPCI Act was an amendment to the Public Health Service Act to create an abbreviated approval pathway for biological products that are demonstrated to be highly similar (biosimilar) to a Food and Drug Administration (FDA) approved biological product. This Act is similar, conceptually, to the Hatch-Waxman Act and it aligns with the FDA's longstanding policy of permitting appropriate reliance on what is already known about a drug, thereby saving time and resources and avoiding unnecessary duplication of human or animal testing [37].

**Figure 2:**
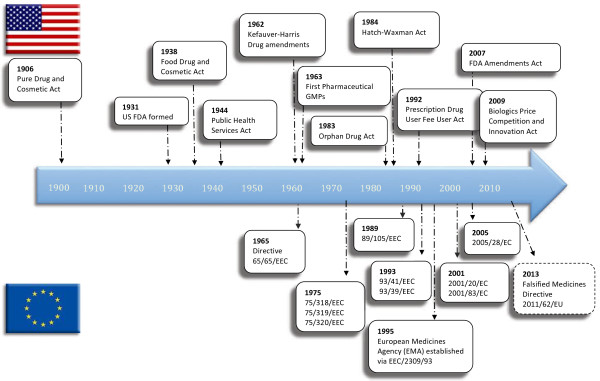
**History of Pharmaceutical Regulations – Timeline of Significant Legislations in the 20**^**th **^**and 21**^**st **^**Centuries.**

Other pieces of legislation have been, and continue to be, enacted to refine aspects of pharmaceutical manufacturing and good manufacturing practices, in addition to ensuring that modern scientific practices and developments are incorporated into law. Refer to Figure 2, **History of Pharmaceutical Regulations – Timeline of Significant Legislations in the 20**^**th**^**and 21**^**st**^**Centuries,** for a schematic timeline of the introduction of the major pieces of pharmaceutical regulatory legislation in the US and EU.

### Major Pharmaceutical Legislation in the European Union

The first European pharmaceutical directive, 65/65/EEC, was brought into force on 26 January 1965 [38]. It aimed to establish and maintain a high level of protection for public health and required prior approval for marketing of originator medicinal products. Much of the impetus behind Directive 65/65/EEC stemmed from determination to prevent a recurrence of the thalidomide disaster in the early 1960s, when thousands of babies were born with limb deformities as a result of their mothers taking thalidomide as a sedative during pregnancy. This experience, which shook public health authorities and the general public made it clear that, to safeguard public health, no medicinal product must ever again be marketed without prior authorization [39].

A decade later, two landmark Directives 75/318/EEC [40] and 75/319/EEC [41] sought to bring the benefits of innovative pharmaceuticals to patients across the European Community by introducing a procedure for mutual recognition, by Member States, of their respective national marketing authorizations. To facilitate mutual recognition, Directive 75/319/EEC established a Committee for Proprietary Medicinal Products (CPMP), which first assessed whether candidate products complied with Directive 65/65/EEC [39].

The Council adopted directives in 1992 on the wholesale distribution, classification for supply, labelling and packaging, and advertising of medicinal products for human use. The EU also introduced pharmacovigilance (the surveillance of the safety of a medicinal product during its life on the market), requiring Member States to establish national systems to collect and evaluate information on adverse reactions to medicinal products and to take appropriate action where necessary.

A new European system for authorising medicinal products came into effect in January 1995 (via Regulation EEC/2309/93 [42] & Directive 93/41/EEC [43]) along with the establishment of the new European Medicines Evaluation Agency. It offered two routes for authorising medicinal products: a "centralised" procedure, through the European Medicines Evaluation Agency (EMEA) (now the European Medicines Agency (EMA) [44]); and a "mutual recognition" procedure through which applications are made to the Member States selected by the applicant, and the procedure operates by mutual recognition of the national marketing authorisation. Additionally, updates to the requirements relating to the placing on the market of high-technology medicinal products, particularly those derived from biotechnology, were put in place.

The newest piece of major legislation in Europe is the Falsified Medicines Directive [2011/62/EU] [45], effective on 02 January 2013, which aims to protect European consumers against the threat of falsified medicines that might contain ingredients, including active ingredients, not indicated on the labelling, are of poor quality or are in the incorrect dose – either too high or too low. As they have not been properly evaluated to check their quality, safety and efficacy they are potentially detrimental to public health and safety. The term 'falsified' is used to distinguish from the infringement of intellectual property rights, so-called 'counterfeits'. As falsifications become more sophisticated, the risk that these products reach patients in the EU increases every year [46].

### Biosimilars

The newest incarnation of off-patent medicines are “biosimilar” medicines, also known as “follow-on biologics”. Biosimilar medicines have been a reality in the European Union for several years and the necessary legal framework was adopted in the EU on 31 March 2004 with the first biosimilar medicines approved by the European Commission in April 2006 [47].

A biological medicine is a medicine whose active substance is made by or derived from a living organism. For example, insulin can be produced by a living organism (such as a bacterium or yeast) which has been genetically manipulated to produce insulin [48]. A “biosimilar” medicine is one that is similar to a medicine of biological origin that has already been authorised (known as the *biological reference medicine*). Biological products are fundamentally different from standard chemical products in terms of their complexity, and it is unlikely that the biosimilar product will have an identical structure to that of the reference product, thereby requiring evidence of safety and efficacy before approval. In this regard, biosimilars are different to the (to date) more familiar generic products.

As with other generic medicines, a biosimilar medicine undergoes testing to ensure that it is as safe and effective as the reference product. However, due to the complex method of production, the active substance may differ slightly between the two medicines and so, additional safety and efficacy studies may be required on a case-by-case basis.

Since biosimilar and biological reference (originator) medicines are similar but not identical, the current recommendation is that the patient should be prescribed the same formulation (either the originator or biosimilar formulation) on each occasion [48]. See Table 1**Examples of Biosimilar Products** (Adapted from [49]) for some examples of originator (i.e. reference) biologics and their biosimilars.

**Table 1:** Examples of Biosimilar Products

**Reference biologic (active substance)**	**Manufacturer**	**Biosimilar products**	**Manufacturer**	**Activity**
Genotropin (somatropin)	Pfizer	Valtropin Omnitrope	BioPartners Sandoz	Human Growth Hormone
Eprex (epoetin alpha)	Johnson & Johnson	Binocrit (epoetin alpha) Retacrit (epoetin zeta)	Sandoz Hospira UK	Control of erythropoiesis
Neupogen (filgrastim)	Amgen	Tevagrastim Filgrastim Hexal	Teva Generics Hexal AG	Granulocyte colony-stimulating factor

### Drug Development

Development of new drugs is a complex and costly process. It generally takes 10–15 years, and studies have shown that it can cost between US$800 million to US$2 billion to get a new drug to market, with similar, or even higher, costs for development of biopharmaceuticals [biologics] [50].

Research and Development [R&D] involves discovery [preclinical studies] and development [clinical studies] of New Chemical Entities [NCEs] also known as New Molecular Entities [NMEs]. It is worth noting that of about 10,000 NCEs investigated to potentially treat a disease, only 250 might make it to animal testing and, of these, approximately 5–10 will qualify for testing in humans. Between 19 and 30% of Investigational New Drugs [INDs] that begin Phase 1 trials make it to marketing [51], meaning that only 1–2 of the original 10,000 NCEs will result in a marketable product.

**Figure 3:**
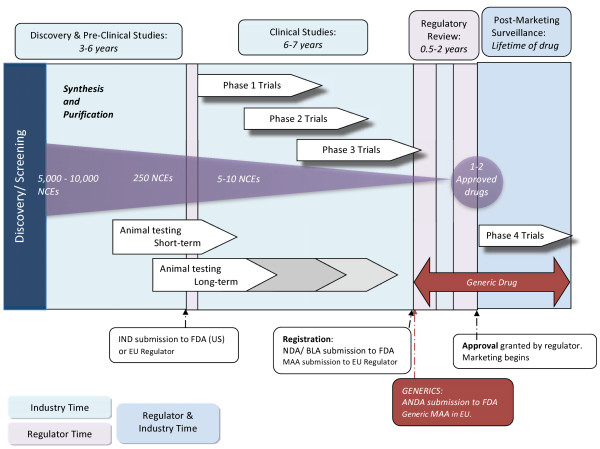
Schematic of Drug Development Process.

An experimental medicine, also known as an Investigational Medicinal Product [IMP], is first tested in *in vitro* laboratory studies and *in vivo* animal studies. Following success here, the testing can move to the clinical phase where the IMP will be used for the first time in human clinical trial volunteers. Refer to Figure 3, **Schematic of Drug Development Process** (adapted from [52]), for an illustration of the process.

### Naming of New Drugs

During the R&D process, a new pharmaceutical substance is given an International Non-proprietary Name [INN] or generic name, in addition to the name that may eventually become its proprietary, or brand, name. Each INN is unique, globally recognised and is public property.

Non-proprietary names are intended for use in pharmacopoeias, labelling, product information, advertising and other promotional material, drug regulation and scientific literature, and as a basis for product names, e.g. for generics. Their use is normally required by national or, as in the case of the EU, by international legislation. As a result of ongoing collaboration, national names such as British Approved Names (BAN), Dénominations Communes Françaises (DCF), Japanese Adopted Names (JAN) and United States Accepted Names (USAN) are nowadays, with rare exceptions, identical to the INN. Names which are given the status of an INN are selected by the World Health Organisation on the advice of experts from the WHO Expert Advisory Panel on the International Pharmacopoeia and Pharmaceutical Preparations.

An important feature of the INN naming system is the use of a common “stem” which indicates the activity of the substance and the pharmacological group to which it belongs. The stem is generally placed at the end of the name, but in some cases it may be placed at the beginning or in the middle of the name. For example: substances having –*adol/-adol-* as the stem indicates an analgesic (e.g. tramadol); *–mab* indicates a monoclonal antibody (e.g. infliximab); *-azepam* indicates a diazepam derivative (e.g. temazepam) and *–vir* indicates antiviral agents (e.g. acyclovir). All of the stems recommended by the WHO are contained in the “stem book” along with guidance for their use [53]. The INN, containing the common stem, provides a single, unique name which enables healthcare professionals to recognise the substance and the family of similar pharmacological substances to which it belongs. The INN is generally the name under which the generic from of a drug is marketed.

### Pre-Clinical Research

The earliest stage of development of a new drug begins with the synthesis and purification of the new chemical moiety, or the screening of existing compounds for potential use as drugs. The aim of pre-clinical research is to determine whether the drug is reasonably safe for potential use in humans, and sufficiently effective against a disease target in chemical tests or animal models. During pre-clinical studies, the pharmacology of the new drug in addition to its pharmacokinetics: absorption, distribution, metabolism, excretion & half-life; and pharmacodynamics: mechanism of action and estimates of therapeutic effects, are assessed. Initial studies relating to toxicology including carcinogenicity, mutagenicity, and teratogenicity are also carried out, as are efficacy studies on animals.

### Clinical Trials

Once permission has been received from the appropriate regulator to administer a new drug to humans, clinical studies may commence. Clinical studies required to bring a new drug to market generally take place over three phases as follows:

· Phase 1: Safety studies on healthy volunteers. Typically involve 20–80 healthy volunteers (women of childbearing potential are excluded). The emphasis is on drug safety and on the building of a safety profile for the drug in humans.

· Phase 2: Clinical studies on a limited scale to determine efficacy of the drug. Typically involve 100–300 individuals who have the target illness. Patients receiving the drug are compared to similar patients receiving a placebo or another drug, and safety evaluations continue.

· Phase 3: Comparative studies on a large number of patients. Typically involve 1000–3000 patients. The emphasis is on safety and effectiveness and studies investigate different populations and different dosages as well as evaluating the new drug in combination with other drugs. Data gathered in a phase 3 trial are used to determine the risk versus benefit profile of the drug.

Following successful completion of clinical trials, the entirety of the information about the drug is compiled into an application and submitted to the relevant competent authority [e.g. FDA in the US, or EMA in Europe]. The competent authority reviews this application, and additional information may be sought from, or discussions held with, the applicant before the regulator makes its decision. The regulator will, after assessing the scientific data pertaining to the new drug, either allow it to be marketed, or deny approval to the applicant.

### Registration

The next step in bringing a new medicine to market is the filing of an application with the health regulatory authority of a country in order to obtain approval to market the new medicine. This step is known as registration. In the US, a New Drug Application (NDA) or Biologic Licence Application (BLA) is filed with the US Food and Drug Administration (FDA). In Europe, a Marketing Authorization Application (MAA) is filed with the European Medicines Agency (EMA), or local competent authority, dependent on the approval route being used. A description of the medicine's manufacturing process along with quality data and trial results are provided to the health regulatory authorities in order to demonstrate the safety and effectiveness of the new medicine. If approval is granted, the new medicine can be marketed for use by patients.

### Post-Marketing Surveillance

Post-market surveillance studies [also called Phase 4 trials] of the drug continually assess the safety of the drug in the marketplace. This may include reporting and investigation of the incidence and severity of rare adverse reactions, cost-effectiveness analyses, comparative trials, and quality of life studies.

### Where Do Generic Medicines Fit Into This Process?

Applications for marketing approval of generic medicines [i.e. the submission of an ANDA in the US, or a generic MAA in Europe] are made at approximately the same time point as the registration step for originator (i.e. proprietary) medicines. Generic medicine applications do not need to contain the pre-clinical and clinical studies required for originator medicines, with relatively simple bioequivalence studies being acceptable in their place, as discussed earlier [8,16].

Referring to Figure 3, it can be seen that the difference in the cost of generic medicines is primarily due to the fact that investment in generics is significantly less than for new originator medicines. Without the need to recoup the costs of pre-clinical and clinical studies, generic manufacturers can price their product lower than the originator product. However, as referred to previously, the market price of the product can be considerably influenced by end-user and prescriber perception, local regulations and reimbursement models [9].

From a production point of view, however, the cost of manufacturing an originator or a generic will probably not differ significantly as they are both manufactured under the same industry standards and conditions. In fact, it is not uncommon for the manufacturer of an originator product to become a manufacturer of a generic version of the drug, once the patent for the drug has expired and it becomes open to generic competition [2]. From an economic perspective, this allows the company to continue recouping the cost of their capital and R&D investment from first introducing the product to the market.

### Are Generic Medicines Really The Same As The Originator Medicines?

It has been clearly shown that, at least at a physiological level, generic medicines behave very similarly to their originator counterparts. As described earlier, an assessment of 12 years of bioequivalence data submitted to the FDA, comparing 2070 single-dose clinical bioequivalence studies of orally administered generic medicine products approved by the Food and Drug Administration (FDA) from 1996 to 2007, demonstrated that the products did not differ significantly [14]. Similarly, referring to clinical efficacy, Kesselheim *et al.* (2008) published an extensive systematic review and meta-analysis (referred to previously) that were favourable towards use of generic drugs in treating cardiovascular disease [13]. In another study they reported that, for anticonvulsant drugs, “evidence does not suggest an association between loss of seizure control and generic substitution” [54]. Further, many studies have demonstrated that initiation of treatment with generic medicines or switching to generic medicines are not associated with poorer patient outcomes. Specifically, Amit *et al.* in 2004 showed that a generic formulation of propafenone, used to treat atrial fibrillation, was found to be at least as safe as the originator drug [55]. Additional evidence of safety in use of generic antipsychotic medicines was provided by Araszkiewicz *et al.*[56] while the safety and efficacy associated with switching of drugs was has also been positively reported [57,58].

Researchers have, however, also reported patient concerns related to generic medicines. These studies range from qualitative assessment of perceptions in specific patient cohorts [59] to general lay/consumer knowledge [60,61], versus knowledge of professionals [62,63]. Many of these studies focus on the influence of relative cheapness on perceptions and use of generic medicines [64,65]. Some publications have shown that consumers felt that a generic medicine did not work either as effectively, or at all, in comparison to when they were taking the originator medicine [66]. For example, reports from patients show that symptoms of depression, which returned while taking a generic medicine, abated again when they switched back to the originator medication [66]. Researchers investigating the efficacy of generic bisphosphonates in the management of osteoporosis demonstrated that they resulted in poorer increases in bone mineral density than branded products [67] and postulated that reasons for this may include higher levels of gastrointestinal adverse events and poor tolerance of generic formulations associated with an increased likelihood of generic particulate matter adhering to esophageal mucosa [68]. However, newly recommended criteria for the evaluation of treatment failure in osteoporosis may stimulate further research into this clinical challenge [69].

In treatment of epilepsy, significant problems have been reported, including breakthrough seizures and increased side effects following a switch to a generic antiepileptic drug [AED] [70]. Additionally, Jain (1993) ascertained that 26 of 131 cases of carbamazepine failure reported to the drug maker were associated with seizure increases following a switch to a generic formulation. Seizure control returned to baseline when the brand formulation was reinstituted [71]. Mayer *et al.* (1999) compared patients who were receiving a generic extended-release carbamazepine formulation with patients taking a branded formulation, in an unblinded trial, and found that 9 of 13 subjects experienced adverse effects when on the generic formulation, with AUC fluctuations that are acceptable within current FDA guidelines [72]. It can be concluded, therefore, that at least in the case of AEDs, bioequivalence, as defined in regulations, does not always correspond to therapeutic equivalence because of the permitted range, evaluation methods and individual variation [73], although this has been refuted in an extensive meta-analysis by Keselheim et al. [54] who found that generic substitution had no impact on efficacy of seizure control.

Incidences such as those described above have resulted in caution being expressed by professionals regarding the safety and effectiveness of generic medicines, albeit in a minority of situations [74,75]. This is contrary to the fact that there is strong evidence that consumers believe that generics are less expensive (and therefore better value) than brand-name drugs, and are as safe [76]. Indeed, the debate is further fuelled by incisive systematic reviews of the published literature. For example, Talati *et al.* (2012) assessed the efficacy, tolerability, and safety of innovator versus generic antiepileptic drugs, and demonstrated that (albeit with a low strength of evidence) initiating treatment with an innovator or generic antiepileptic drug will provide similar efficacy, tolerability, and safety but that switching from one form of medication to the other may be associated with more hospitalizations and longer hospital stays [77]. Some experts have stated that switching between originator and generic drugs may actually be unethical, raise the cost of treatment, with additional clinic visits and laboratory tests [78,79]. Similar arguments are made when addressing the concept of therapeutic substitution, whereby there may be an attractive price differential between established drugs whose patents have expired and for which generics are available and newer (or branded) medicines within the same therapeutic class, as researchers have made the point that direct evidence to support equivalence may be lacking [80-82].

While the active pharmaceutical ingredient (API) does not differ between originator and generic medicines, other (inactive) ingredients, known as excipients, may be different and a number of pharmaceutical excipients are known to have side effects or contraindications [83]. As excipients may differ between originator medicines and generic preparations which have been shown to be bioequivalent and therefore substitutable, there needs to be an awareness in the medical/healthcare community that where a generic preparation contains an excipient which is not part of the originator preparation, there is the potential for the generic formulation to cause problems in a patient who had no issues in tolerating the original preparation.

Evidence has been published that differences in excipients between originator medications and their generic counterparts can cause problems. For example, allergic reaction has been reported to croscarmellose sodium used as excipient in a generic furosemide preparation in a patient who had previously been taking branded furosemide without incident [84]. (Croscarmellose is used in injectable preparations as a suspending agent to promote solubilization of compounds with poor water solubility; it is also present in tablets as binder, glidant and antiadherent).

Similarly, a lactose-intolerant patient with an arrhythmia who is switched from one formulation of antiarrhythmic drug to another that contains a lactose-based excipient may experience gastrointestinal disturbances which could affect gut transport time and overall drug absorption, thereby affecting systemic levels of the drug [85]. Studies have also reported significantly different serum levels of antiarrhythmic drugs associated with originator products and their generic equivalents, in addition to observing patients’ symptoms recur following a switch to a generic formulation. These observations have led to the conclusion that there is evidence that formulation substitution in the cardiovascular arena has risks [85].

More broadly, allergies to excipients contained in topical steroids have also been well documented [86] - with these allergens being contained in both originator and generic preparations. Saccharose, an excipient with potential side effects, was seen in generic preparations of phenobarbitol used to treat epilepsy in Mauritania [87]. Lactose and saccharose are contraindicated in people with lactase or saccharase deficiencies and as the frequency of these enzyme disorders is high in African populations [88], this suggests the potential for negative clinical reaction to such medicines in African patients.

Therefore, while bioequivalence between an originator medicine and a generic equivalent may have been proven, as required by the current regulatory guidelines, given the differences in other ingredients it is incumbent on prescribing physicians to remain vigilant to the potential risks, and exercise caution in the substitution of a medication with an equivalent. This is applicable to both substitution of a branded medication with a generic equivalent and to switching between different, equivalent, preparations of generic medications (e.g. the same generic medication produced by different manufacturers).

This, however, is not an effect limited to use of generic medicines. As described earlier, Patel *et al.* reported that (in 2010) patients prescribed an anti epileptic medication experienced unexplained toxicity [15] which, when investigated, was found to be due to altered formulation.

Despite this, regulators have, in some cases, adopted a cautious approach in legislating for potential risks associated with generic substitution, in particular possible challenges relating to continued efficacy and safety of treatment under defined circumstances. In July 2011, the Danish Government banned generic substitution for immunosuppressants (specifically, cyclosporine and tacrolimus) due to issues relating to the possible need for increased testing requirements following use of generics in transplant patients [89]. Similarly, the British National Formulary (BNF) currently recommends brand prescribing for a number of medicines and drug classes, namely modified release diltiazem [90] p132]) and cyclosporine [90] p583], while in July 2008 the Northern Ireland Health and Social Care Board issued an extensive list of medicines considered unsuitable for generic prescribing [91] which included narrow therapeutic index drugs, modified release preparations, controlled drugs including patches, inhalers, and multi-ingredient products.

### Usage of Generic Medicines in Ireland, a Case Study

Irish healthcare spending in 2010 accounted for 9.2% of GDP [92], with total expenditure on pharmaceuticals amounting to €2.2 billion, and public expenditure on pharmaceuticals (administered by the Primary Care Reimbursement Service, PCRS) amounting to €1.9 billion. Public expenditure on pharmaceuticals was one of the fastest growing components of public health expenditure over the period 2000 to 2010. It increased by 158.5 per cent in real terms and accounted for 12.9 per cent of total public health expenditure in 2010 (up from 10.1 per cent in 2000) [92].

State assistance towards the cost of pharmaceuticals is available under a number of different schemes. The General Medical Services (GMS, or medical card) Scheme provides free public health care (including GP care and prescription pharmaceuticals) to those who satisfy an income means test. In April 2011, over 1.6 million individuals had a medical card, accounting for 36.2 per cent of the population. A further 2.6 per cent of the population were eligible for free GP services (but not prescription pharmaceuticals) under the GMS Scheme (known as GP Visit card holders) [93]. Non-medical cardholders avail of State assistance towards the cost of prescribed pharmaceuticals under a number of Community Drugs Schemes (CDS). The three largest (in expenditure terms) are the Drugs Payment (DP), Long Term Illness (LTI) and High Tech Drug (HTD) schemes. At the time of writing, all those ineligible for a medical card were eligible for the DP Scheme, whereby the State pays the full cost of prescription pharmaceuticals and certain appliances above a monthly threshold of €132 per family.

**Figure 4:**
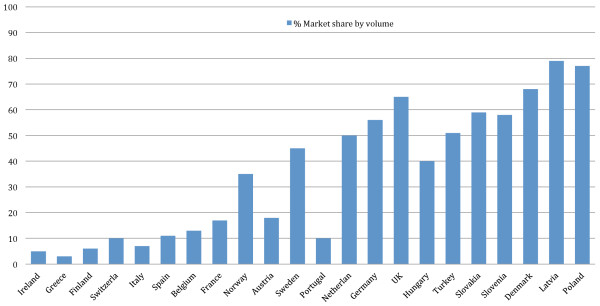
Market Shares (By Volume) of Generic Medicines in Europe in 2006 (reproduced with permission from the European Generic Medicines Association).

Penetration of generic medicines into the Irish market is amongst the lowest in Europe. See Figure 4: **Market Shares (By Volume) of Generic Medicines in Europe in 2006** (data from [94]). Furthermore, in a report written by the NCPE for the Irish Department of Health and Children (DOHC) in 2008 it was reported that generic prescribing in Ireland had fallen from over 22% by volume in 1997, to just over 19% in 2007 [93]. As a result of this poor penetration by generic medicines, Irish expenditure per 1000 inhabitants per annum is ten times that of Sweden, putting in perspective the considerable need to quickly realize the substantial savings that are possible without compromising patient safety or efficacy of treatment.

In 2008, approximately a quarter of all prescriptions dispensed on the GMS, DP and LTI schemes had an available generic equivalent [95]. That translated into €227.76 million which was spent on originator medicines where there was an equivalent, less expensive, generic product available representing a potential area for cost saving to the Irish state. Moreover, from the NCPE’s 2008 figures, an immediate opportunity for increase in use of generics in Ireland can easily be seen, as the DP/LTI figures show a difference of 7% in the proportion of prescriptions that were for generics compared to generic prescriptions from the GMS [96].

### Cost-Saving Potential in Ireland

The net cost of the Irish Community Drug Schemes more than doubled in a seven year period, from €1.024 billion in 2001 to more than €2.289 billion in 2007 [96]. The increase is partly explained by a “low” price fixed in 1992, subsequently renegotiated, and 50% mark-up (on ingredient costs) and dispensing fees agreed with dispensing pharmacists in parallel with an ageing population accessing the GMS scheme. The four schemes together account for 98% of prescriptions and 99% of expenditure in the community setting [97].

Cost-saving opportunities, including something as straightforward as closing that 7% difference between GMS and DP/LTI generic prescription figures, may be critical to the Irish state, in an era when healthcare costs are escalating. Furthermore, it was reported in 2003 that there was the potential for 40% of medicines prescribed on the GMS to be dispensed generically [98]. As increasing numbers of originator medicines reach the end of their patent/exclusivity periods, thereby allowing generic competitors to enter the market, this area represents an increasing potential for cost savings for the public purse.

Total expenditure on originator medicines in Ireland rose from €120 million in 2004 to more than €220 million in 2008 [99]. In 1997, the average cost per dispensed item under the GMS scheme was €11.20 as compared with €23.27 in 2007. Factors contributing to the increase in drug expenditure include the “product mix” (the prescribing of newer, more expensive medications) and the “volume effect” (comprising of the growth in the number of prescription items issued). In 1997, twenty million prescription items were issued under the GMS scheme. This increased over two-fold to 44.35 million items in 2007. The year on year increase in pharmaceutical expenditure in Ireland is amongst the highest in Europe with medicines, in 2009, accounting for approximately 13.5% of total healthcare spending [100].

Healthcare costs, as previously demonstrated, may be somewhat mitigated by increased prescription of generic medicines. It has been recommended that the State examine the price it pays for generic medications and encourage greater INN prescribing by doctors [95]. In recent years, a trend towards the prescription of generic medicines has been seen worldwide. For example: in the US, in 1984, only 14% of prescriptions were for generic medicines. This had increased to 66% in 2006 [95] and by 2011 78% prescriptions written in the US were for a generic medicine [8]. In the UK, generic medicines accounted for approximately 83% of all prescriptions written in 2009 and 2010 [101]. [That rate, incidentally, is generally considered to be in the region of the maximum expected rate of generic prescribing]. A key driver for such a high rate of generic prescribing has been the training of UK physicians to prescribe by INN where possible, something which is subsequently continued and encouraged in practice [102] and is one of multiple initiatives used in Scotland with particular emphasis on PPIs and statins [103]. It is worth noting that in Ireland, in 2007, generic prescribing comprised 2.6% unbranded generics versus 16.4% branded generics (78) and is, thus, a target for education regarding generic usage. Indeed, a related aspect of branded versus unbranded generic prescribing is that there is evidence of confusion where patients are dispensed a different branded generic on each pharmacy visit, resulting in pharmacist (and, presumably, physician) resources being invested in explaining to the patient that their new drug is the same as the previous one [104]. A lesson for Ireland may be that such patient confusion could be avoided if related education of patients is introduced with implementation of the new policy.

An increase in use of generics is associated with significant cost savings, for example in 2010 alone, the use of generics in the American health system saved $158 billion, an average of $3 billion every week [25] and a study by the Generic Pharmaceutical Association (GPhA) showed that prescribing of generics has saved the US economy $931 billion between 2001 and 2010 [105].

In mid 2010, the Irish Minister for Health announced plans to introduce new legislation to allow the introduction of reference pricing and to permit generic substitution/medicine interchangeability in Ireland. A report entitled the *Proposed Model for Reference Pricing and Generic Substitution,* which describes the model to be implemented in Ireland, indicates the reason for the proposed changes:

*Demographic changes over the next decade will have a significant impact on the demand for and the delivery of health care in Ireland. Pharmaceutical expenditure accounts for a large proportion of overall health care expenditure. In 2008, the Health Service Executive paid for approximately 65 million prescription items at a cost of over €1.9 billion. As a result of demographic changes and prescribing trends, the number of prescription items is estimated to increase to 105 million by 2021 at cost of €2.4 billion. The current system is unsustainable. To ensure that patients can continue to access innovative and affordable medicines, new pricing and reimbursement approaches are required, along with changes in prescribing practices*[106].

At the time of writing, the current situation in Ireland requires that the pharmacist supply only the medicine indicated by the prescribing physician, even if there is a cheaper, generic version available. The new legislation would require the dispensing pharmacist to notify the patient/customer if a lower-priced, (probably generic) alternative were available, and to allow that alternative to be dispensed. It seems reasonable to suggest that, given the success of INN prescribing in the UK [107] that its adoption by physicians practicing in Ireland would complement the pharmacy-based approach described above.

### Reference Pricing & Generic Substitution

With reference pricing, a common reimbursement price or reference price, is set for a group of interchangeable medicines based on the price of a “reference drug” which is chosen from that group of drugs [25,103]. The reference drug will be as safe and effective as the other available drugs in the group and may or may not be a generic medicine. The price of this reference drug is the price paid by the State, and if the patient/consumer wishes to have a different, more expensive, drug to the reference drug, they must pay the difference in price themselves [107]. Provision is generally made for prescribers to prohibit substitution for clinical reasons. In these instances, patients do not face any additional costs if the prescribed product costs more than the reference price.

At the time of writing, neither generic substitution nor reference pricing are permitted in Ireland despite being used in many other countries both in Europe and elsewhere. In these countries, the pharmacist can substitute medicines that have been designated as interchangeable – that is: a medicine of the same quality and clinical efficacy, but of a lower cost, can be dispensed in place of what was prescribed.

However, despite evident success in a number of countries, it has been argued that additional savings may be possible without impacting the continued efficacy or safety of patient treatment. A 2007 study by Kanavos [108] reported that the UK National Health Service was reimbursing for generic medicines at too high a price, and that a considerable proportion of the reimbursed price accrued to the distribution chain in a fashion that resembles standard retail models. Indeed, it was claimed that this overpayment effectively constituted a subsidy to pharmacists (intended or otherwise). Analogous overpayments were reported in a study of pharmacy discounts in France [109] where control of pharmaceutical expenditure has been a national policy priority for many years and health system measures have included reference pricing, generic substitution and international non-proprietary name (INN) prescribing. However, as in other markets, generic manufacturers and wholesalers offer discounts, rebates or promotions to pharmacies to gain an advantage over competitors, meaning that health insurance in France may be overpaying for generic medicines. As Ireland moves towards a formalized generic medicine policy, an opportunity presents itself to ensure the reimbursement costs are close to market price (including savings associated with volume discounts referred to earlier) and that the benefits of the new policy do not accrue disproportionately to the pharmacists and their wholesalers and medicine distributors.

### Concerns

While the main objective for the introduction of generic substitution and reference pricing is to reduce costs related to healthcare for both the consumer and the State, the concept of reference pricing is not without its concerns.

The Irish Pharmacy Union (IPU) warned that reference pricing could lead to shortages of medicines and the Irish Pharmaceutical Healthcare Association (IPHA) stated that Ireland currently has a fair and equitable single-tier system whereby all patients, regardless of income, have access to secure supply of the medicines which their doctors believe are most suitable for them [110,111]. The IPU and IPHA believe that should the Health Services Executive [HSE] set the reference price at that offered by the lowest potential supplier, it could give rise to patients being dependent on one supplier, which could, perhaps, have very limited infrastructure or commitment to the Irish market. This, however, seems at odds with the market situation whereby smaller countries (such as Lithuania which has a population comparable to Ireland) obtain sufficient supplies of products including generic medicines at considerably reduced prices [112] and may, actually, represent an aversion to erosion of profits rather than accurately reflecting the market.

Concerns have also been expressed by organisations such as the Irish Medical Organisation (IMO) and Irish College of General Practitioners (ICGP). During a working group meeting held in January of 2010, the ICGP cautioned that switching of medicines may not be suitable and also that when determining which products are substitutable “the tests of bioequivalence must be robust” [113]. This point regarding equivalence is, as referred to previously, equally relevant to variability between successive batches of originator or generic products.

### Acceptance of INN prescribing and Substitution by Prescribers in Other Countries

When generic substitution was first introduced in Australia (the Brand Substitution Policy (BSP); introduced in December 1994 [114]), two studies were conducted exploring medical practitioners’ views on generic medicines and generic substitution. The first study, conducted in 1995, five months after the government permitted generic substitution by pharmacists, was a national telephone survey of GPs. Out of a total of 71 GPs, 28 (39%) said ‘no’ to generics substitution, 22 (31%) said ‘yes’ to substitution and 21 (30%) were ambivalent [115]. The most common reason cited by those opposed to generics prescribing and substitution was that it would cause confusion among patients, particularly the elderly, because generic brands were often of different colours and shapes. (This argument ignores the fact that packaging and presentation of originator medicines may also differ depending on country of origin if sourced via parallel importation). Other reasons given were that it was a doctor’s responsibility, not a pharmacist’s, to decide on medication and that using generics meant less money for research. There were also concerns about bioavailability, adverse reactions to generics and the need for a free enterprise environment [115]. Despite presumably greater familiarity with generic medicines, similar views were expressed by some of the doctors in the second study, which was conducted in 2002 [115].

Analogously, in Sweden, researchers saw that while generic substitution was implemented in 2002, only 60% of the possible indicated savings were made in the first year, due somewhat to the mix of generic and originator products stocked by pharmacists [116]. Subsequent studies by Anderssen *et al.* showed that gender and age influenced Swedish patients’ generic medicine use [117] and further documented rational use of generic medicines through the establishment of drug and therapeutic committees, development of guidelines, academic detailing, continuous benchmarking of prescribing patterns and financial incentives that have led to effective implementation of a generic medicine policy by stakeholders recognizing the need to conserve resources [118].

In the US, individual physicians have expressed strong opinions about generic medicines over the years, with opponents of their use generally being more vocal. In 1997, Banahan and Kolassa [119,120] reported a comprehensive analysis of physicians' attitudes toward generic medications, finding that overall, physicians' attitudes toward generic medicines were fairly neutral, as indicated by their answers to two key questions from their nationwide survey. In a separate study (from 2001), respondents expressed modest support for generic substitution, but had doubts about originator-generic equivalence [121]. More recently, Shrank *et al.* have reported studies addressing the relationship between generic medicine prescribing and physician practice location and specialty (i.e. higher income catchment areas equated to higher generic prescribing rates and generalist physicians prescribed more generic medicines than specialist physicians) [122]. Shrank *et al.* have also shown that persistence in generic medicine use is higher than with branded products in those patients benefitting from incentives offered by medical insurance companies and pharmacy drug purchase plans [123]. Perhaps most interesting is that, in 2012, Shrank *et al.* found that a meaningful proportion of physicians expressed negative perceptions about generic medications, representing a potential barrier to generic use. The researchers recommended that policymakers trying to encourage generic use should consider educational campaigns targeting older physicians [123].

Similar results were seen in a study carried out amongst Irish prescribers in 1997, which showed that the majority of prescribers were concerned about the reliability and quality of generic medicines [75], and the study concluded that education of stakeholders would be necessary to improve the level of INN prescribing in Ireland. An additional survey in 1997 indicated that over a third of Irish GPs believed that generic medicines were unreliable and of poor quality and 50% of pharmacists believed that some generic medicines were unreliable [124].

The United Kingdom consistently has higher rates of generic prescription than Ireland, and this is generally thought to be due to the fact that Government policy in the UK actively promotes INN prescribing from medical education to subsequent ongoing practice (as described earlier) through processes of monitoring or prescribing of generic versus originator/patented products. The introduction of fundholding practices provided further encouragement from a financial perspective [125], whereby medical practitioners’ fixed budgets provided an explicit incentive to contain costs, which in turn encouraged INN prescribing.

### Previous Attempt at Improvement of Use of Generic Medicines in the General Medical Services Scheme in Ireland

In 1993, a drug budgeting arrangement called the Indicative Drug Target Savings Scheme [IDTSS] (also referred to as the Indicative Drug Budgeting Scheme) was introduced in Ireland as a result of a voluntary agreement entered into with the Irish Medical Organisation and the Department of Health [124]. The purpose of this scheme was to curtail spiraling GMS prescription costs by encouraging rational and cost-effective prescribing [125,126]. With this agreement, an annual “indicative drug budget” was calculated for each participating GMS GP, based on a combination of the doctor's previous prescribing costs and the national average [126].

Typically, 50% of any savings made on these indicative drug budgets, achieved primarily through use of increased prescribing of generic medicines, were returned to the prescribing physician [127]. All savings had to be invested in the development of the general practitioner’s own practice. There were no penalties for overspending.

A report reviewing the IDTSS in 1997 indicated that the scheme saved IR£13.5 million [i.e. €17.14 million] during 1993–1994 [128]. Additionally, it showed that even those prescribers who exhibited lower-cost and fewer-item prescribing per patient, prior to implementation of the scheme, were successful in reducing their cost per item further through increased use of generic prescriptions [125]. The main conclusions from this report included that there were changes in prescribing behaviours, seen as enhanced prescribing of generic medicines, leading to lower drug costs per patient. Also, there were no discernible negative effects on overall quality of prescribing observed.

A study of the IDTSS by Walley *et al.* showed that the IDTSS encouraged changes in prescribing practice among low and medium cost prescribers, but had no apparent effect in higher cost prescribers. The changes were relatively short-lived with a similar rate of rise of costs across all groups by the third year of the scheme (1996) [125]. This is broadly similar to the effects of GP fundholding in the UK, where new fundholders dramatically reduced their prescribing costs, but where there were similar rates of rise of prescribing costs after 2–4 years in both fundholding and non-fundholding practices [127].

Despite the reported savings in the first year of the scheme [IR£13.5 million in 1994 [129]], the scheme was not entirely successful; 27% of GPs never achieved any savings in the first 4 years of the scheme [125]. Since December 2005, however, a freeze was placed on this scheme [127]. This may have a factor in the previously mentioned fall in prescribing of generic medicines seen between 1997 and 2008 in Ireland.

## Conclusions

While acceptance of the definition of a generic medicine is ostensibly similar worldwide, there are some discrepancies between different jurisdictions, particularly related to determination of bioequivalence. For example, Narrow Therapeutic Index drugs have distinct and different bioequivalence acceptability in the EU that is not in place in the US [126]. Differences such as this, in addition to the fact that components of a generic medicine [with the exception of the API], the appearance of the medicine and its packaging, can differ between apparently equivalent originator and generic medicines have led to publication of reports describing variability in efficacy and adverse events [130]. However, in a balanced debate, these studies and expressions of distrust should be evaluated alongside the many reports that have demonstrated comparable effectiveness and acceptability between generic and originator medicines [66,67,74,75].

Researchers have explored the attitudes and beliefs of stakeholders in the medicines process (that is: prescribers, dispensing pharmacists and patients/end users) demonstrating a spectrum of perceptions and opinion which are influenced by factors such as geography, age and demographics [54-56]. This information clearly demonstrates that if countries are to take advantage of the apparent economic benefits associated with generic medicine use, at least some of their “demand side” [118] activities should focus on education and enforcement to address the excessively sceptical perception of these products. Such approaches to enhance adoption of generic medicines should also be complemented by in-depth analysis of the potential disadvantages of generic products, such as potential variation in quality and formulation [25-29] and associated effects. However, it is probable that existing pharmacovigilence/surveillance systems, which are in place for all human medicines, will be sufficient for monitoring of these. It cannot, however, be disputed that the reputation and perception of reliability of generics needs improvement in the eyes of those healthcare professionals and patients who have articulated poor opinions of them.

The economic benefits of the use of generic medicines cannot be denied; and in many countries their use is essential to control healthcare spending. Given that the majority of patient-doctor encounters result in the writing of a prescription [131], the cost of the medicine prescribed is of interest both to the patient/consumer and the State. The potential cost savings associated with the use of generic medicines must be considered by the bill-payers. In this paper, we have focused on Ireland’s emerging policy on generic medicine use. With Ireland now poised to make the legislative changes required in order to take advantage of generic substitution and ref-erence pricing, the onus is on Ireland’s Health Service Executive, as well as the prescribers and dispensers of medicines, to ensure that they are fully informed of all the complexities associated with the use of generic medicines.

It is also important to learn from lessons of the past and to take into account previous attempts at increasing prescription rates of generic medicines in Ireland, such as the Indicative Drug Target Savings Scheme [IDTSS], as described earlier. Additionally, the Irish government and policy makers may find it useful to look at how generic substitution was successfully implemented in other countries and to take advantage of the depth of information and research available from other jurisdictions which have previously adopted generic substitution and reference pricing. It is in the best interests of the Irish healthcare system for its leaders to learn from the successes and challenges that have already been experienced by other countries such as the UK, France, Germany, Sweden and Lithuania, amongst others. In doing so, education of all stakeholders, including physicians and allied professionals and, in particular, end-users will be pivotal for the appropriate implementation and acceptance of policies for generic substitution/medicine-interchangeability and reference pricing in Ireland.

With many medicines hitting the so called “patent cliff”, generic drug usage, already trending upwards, is likely to continue to increase in the coming years, with generic medicines now being, primarily for economic reasons, a reality of modern healthcare systems.

## Endnotes

^a^For the purposes of this article, the terms “generic drug” and “generic medicine” are considered interchangeable, and therefore, for simplicity of language, only the term “generic medicine” is used throughout.

## Competing interests

The authors declare that they have no competing interests.

## Authors’ contributions

SD researched and wrote this review. WC, BS and CD provided guidance, critical review and revision of the manuscript. All authors read and approved the final manuscript.

## Authors’ information

SD - B. Sc. (Hons), M.Sc.: PhD Candidate with the Graduate Entry Medical School, University of Limerick, Ireland.

BS - MD, FRCGP, MICGP: Director of International Liaison, Graduate Entry Medical School, University of Limerick, Ireland.

CD - BSc, PhD, MBA: Chair & Director of Research and Director, Centre for Interventions in Infection, Inflammation & Immunity (4i), Graduate Entry Medical School, University of Limerick, Ireland.

WC - MD, MICGP, MRCGP, H. Dip.: Professor of General Practice, Graduate Entry Medical School, University of Limerick, Ireland.

## Pre-publication history

The pre-publication history for this paper can be accessed here:

http://www.biomedcentral.com/2050-6511/14/1/prepub
